# Three Minutes of All-Out Intermittent Exercise per Week Increases Skeletal Muscle Oxidative Capacity and Improves Cardiometabolic Health

**DOI:** 10.1371/journal.pone.0111489

**Published:** 2014-11-03

**Authors:** Jenna B. Gillen, Michael E. Percival, Lauren E. Skelly, Brian J. Martin, Rachel B. Tan, Mark A. Tarnopolsky, Martin J. Gibala

**Affiliations:** 1 Department of Kinesiology, McMaster University, Hamilton, Ontario, Canada; 2 Department of Pediatrics and Medicine, McMaster University, Hamilton, Ontario, Canada; Tokyo Institute of Technology, Japan

## Abstract

We investigated whether a training protocol that involved 3 min of intense intermittent exercise per week — within a total training time commitment of 30 min including warm up and cool down — could increase skeletal muscle oxidative capacity and markers of health status. Overweight/obese but otherwise healthy men and women (n = 7 each; age  = 29±9 y; BMI  = 29.8±2.7 kg/m^2^) performed 18 training sessions over 6 wk on a cycle ergometer. Each session began with a 2 min warm-up at 50 W, followed by 3×20 s “all-out” sprints against 5.0% body mass (mean power output: ∼450–500 W) interspersed with 2 min of recovery at 50 W, followed by a 3 min cool-down at 50 W. Peak oxygen uptake increased by 12% after training (32.6±4.5 vs. 29.1±4.2 ml/kg/min) and resting mean arterial pressure decreased by 7% (78±10 vs. 83±10 mmHg), with no difference between groups (both p<0.01, main effects for time). Skeletal muscle biopsy samples obtained before and 72 h after training revealed increased maximal activity of citrate synthase and protein content of cytochrome oxidase 4 (p<0.01, main effect), while the maximal activity of β-hydroxy acyl CoA dehydrogenase increased in men only (p<0.05). Continuous glucose monitoring measured under standard dietary conditions before and 48–72 h following training revealed lower 24 h average blood glucose concentration in men following training (5.4±0.6 vs. 5.9±0.5 mmol/L, p<0.05), but not women (5.5±0.4 vs. 5.5±0.6 mmol/L). This was associated with a greater increase in GLUT4 protein content in men compared to women (138% vs. 23%, p<0.05). Short-term interval training using a 10 min protocol that involved only 1 min of hard exercise, 3x/wk, stimulated physiological changes linked to improved health in overweight adults. Despite the small sample size, potential sex-specific adaptations were apparent that warrant further investigation.

## Introduction

Interval exercise is characterized by repeated bursts of relatively intense effort, interspersed by periods of rest or lower-intensity exercise for recovery. Short-term interval training protocols can induce physiological remodeling similar to continuous moderate-intensity training, despite reduced time commitment and a relatively small total exercise volume [1]. Recent studies have also shown improvements in various health indices including markers of glycemic control in both healthy individuals [2]–[4] and people with cardiometabolic disorders including type 2 diabetes [5] after low-volume interval training. These studies have been conducted on relatively small numbers of subjects and involved relatively short training interventions. Nonetheless, the findings have garnered significant interest from a public health perspective, given one of the most commonly cited barriers to regular exercise participation is “lack of time” [6].

A common interval training model is the Wingate Test, which involves a 30 s “all out” burst of cycling on a specialized ergometer. Typically, 4–6 such intervals are performed, separated by ∼4–5 min of recovery, with three training sessions performed each week [1]. Despite the very small total amount of exercise, a training session typically lasts ∼25 min, given the brief warm-up and cool down that are usually included in addition to the recovery periods. The relative “time efficiency” of Wingate-based training has therefore been questioned [7], considering the ∼75 min time commitment per week, which falls within the physical activity guidelines advocated by some public health agencies. While 150 min of moderate-intensity exercise per week is the general recommendation [8], [9] some guidelines include 75 min of vigorous physical activity as an alternative [9].

Several recent studies investigated physiological and health-related adaptations to very low-volume interval training protocols that involved a time commitment of ≤15 min per session [10]–[12]. For example, Metcalfe and colleagues [10] reported that a 10 min training protocol, involving low-intensity cycling except for 2, 20 sec all out sprints, improved cardiorespiratory fitness (VO_2_ peak) in previously sedentary adults when performed 3x/wk for 6 wk. The potential for very low-volume interval training protocols to improve VO_2_ peak has also been described by Ma et al. [11] and Hazell et al. [12]. Metcalfe et al. [10] also reported that insulin sensitivity based on oral glucose tolerance tests was improved after training in men but not women, highlighting the potential for sex-based differences in the adaptive response. Only one study has examined muscle adaptations to this type of training, with Ma et al. [11] reporting increased protein content of some mitochondrial enzymes after training, although the maximal activity of citrate synthase was unchanged.

The purpose of the present study was to clarify and advance our understanding of the impact of very low-volume interval training on physiological and health related adaptations to very low-volume SIT. Specifically, we examined the impact of a training protocol that involved only 1 minute of intense intermittent exercise within a 10 min time commitment, including warm-up and cool-down. Sedentary but otherwise healthy subjects trained 3x/wk for 6 wk, and needle biopsies were obtained before and after training to examine skeletal muscle remodeling. We also assessed changes in several markers reflective of cardiometabolic health. In light of the findings by Metcalfe et al. [11], a secondary aim was to explore potential sex-based differences in the adaptive response to this type of training. We hypothesized that the training intervention would increase skeletal muscle oxidative capacity, as reflected by the maximal activity and protein content of mitochondrial enzymes, increase VO_2_ peak, and reduce resting blood pressure and 24 h mean blood glucose concentration measured using continuous glucose monitoring (CGM) under conditions of controlled activity and feeding. We further hypothesized that reductions in 24 h glucose would be superior in men.

## Materials and Methods

The protocol for this study and supporting TREND checklist are available as supporting information; see Checklist S1 and Protocol S1.

### Subjects

Fourteen overweight or obese men and women were recruited by poster advertisement from the McMaster University community and took part in the study (Table 1). Subjects were deemed sedentary based on their self-reported habitual physical activity, which consisted of ≤2 sessions/wk of structured exercise lasting ≤30 min. Participants were allocated into the male or female intervention group and matched for age, body mass index and VO_2_ peak. The experimental protocol, which consisted of familiarization and baseline testing, a 6 wk training intervention, and post-training measurements, was approved by the Hamilton Integrated Research Ethics Board and all visits took place at McMaster University. All subjects provided written informed consent prior to their participation.

**Table 1:** Subject Characteristics.

VARIABLE	MEN	WOMEN
Age (y)	29±9	30±10
Height (cm)	176±5	162±8
Weight (kg)	97±8	75±12*
Body Mass Index (kg/m^2^)	31±2	29±2
VO2peak (L/min)	3.0±0.5	2.0±0.2*
VO2peak (ml/kg/min)	31±4	28±4
Maximal Workload (W)	262±30	202±23*

Values are means ± S.D. N = 7 for men and women. VO2peak, maximal oxygen uptake.

*Significantly different from men (p≤0.05).

### Experimental Protocol

#### Familiarization and baseline testing

Participants reported to the laboratory on four separate occasions over 14 d for familiarization and baseline testing during May-July 2013. On the first visit, subjects initially sat quietly for 10 min prior to 3 separate measurements of blood pressure using an automatic blood pressure cuff (Contec 08A, Qinhuangdao, China), with the lowest of these values used for analysis as previously reported [13]. Subjects subsequently performed an incremental maximal oxygen uptake (VO_2_ peak) test on an electronically braked cycle ergometer (Lode Excalibur Sport V 2.0, Groningen, The Netherlands) as previously described [14], [15]. Briefly, following a 2 min warm-up at 50 W, the resistance was increased by 1 W every 2 s until volitional exhaustion or the point at which pedal cadence fell below 50 rpm. A metabolic cart with an on-line gas collection system (Moxus modular oxygen uptake system, AEI Technologies, Pittsburgh, PA) acquired oxygen consumption (VO_2_) and carbon dioxide production (VCO_2_) data. VO_2_ peak was defined as the highest average oxygen consumption over a 30 s period. Approximately 15 min following the VO_2_ peak test, participants performed 1–2×20 s all-out sprints on an electronically braked cycle ergometer (Veletron, RacerMate, Seattle, WA, USA) to become acquainted with the interval protocol.

Approximately 5 d after the familiarization session, participants returned to the laboratory and were fitted with a continuous glucose monitor (CGM; CGMS; iPro, Medtronic, Northridge, CA) and chest-worn accelerometer (Actiheart; Camntech, Cambridge, United Kingdom). Subjects were also given a glucose meter (OneTouch UltraMini, Lifescan, Milpitas, CA) with instructions on how to perform capillary blood sampling. Participants received a standardized food parcel, which they were instructed to consume at prescribed meal times over the subsequent 24 h. The diet was individualized for each participant and energy intake was estimated using the Mifflin-St Jeor equation [16]. Mean total energy was 2623±123 and 1886±146 kcal for men and women, respectively, derived from 56±1% carbohydrate, 30±1% fat and 14±1% protein.

Starting at 600 h the day following CGM insertion, participants began consuming the control diet under free-living conditions and CGM data was collected for a 24 h period. Participants obtained capillary blood glucose samples at four points over the 24 h period when blood glucose was expected to be stable (i.e. upon awakening, before lunch, before dinner and before bed) and were automatically stored in the glucose meter provided. Average blood glucose concentration, glucose area under the curve (AUC) and the daily peak glucose concentration (G_max_) were calculated from CGM data for a 24 h period from 600 to 559 h before and after training. Physical activity was monitored continuously over this 24 h period using a chest-worn device (Actiheart) that simultaneously measured heart rate and activity with an internal accelerometer that senses the frequency and intensity of torso movements to calculate energy expenditure. Following CGM removal at ∼1200 h, glucose data were uploaded as previously described [5].

Approximately 2 d later, participants reported to the lab following a 10 h overnight fast. A single resting blood sample was obtained by venipuncture from an antecubital vein. Plasma and serum were separated by centrifugation (10 min at 4000 rpm) and stored at −20°C for subsequent analysis. A resting skeletal muscle biopsy was obtained using procedures we have previously described [17]. Briefly, muscle samples were obtained from the vastus lateralis under local anesthesia (1% lidocaine) using a Bergstrom needle adapted with suction. Samples were sectioned into several pieces, immediately snap frozen in liquid nitrogen and stored at −80°C for later analysis.

#### Training protocol

At least 5 d following the muscle biopsy, subjects initiated the interval training program, which consisted of 18 supervised sessions over 6 wk during June-August 2013. Training was performed on Monday, Wednesday and Friday each week. Each session consisted of 3×20 s all-out cycling efforts against a load corresponding to 0.05 kg/kg body mass, separated by 2 min of low intensity cycling (50 W), on an electronically braked ergometer (Veletron, RacerMate, Seattle, WA, USA). All training sessions included a 2 min warm-up and 3 min cool-down at 50 W, for a total time commitment of 10 min. The weekly training protocol therefore involved a total of 3 min of very intense intermittent exercise within a time commitment of 30 min including warm-up, cool-down and the recovery between efforts. Peak and mean power output was recorded for each sprint and an average determined for each session. Heart rate (HR) was measured continuously on the first training session.

#### Post-testing

**Figure 1:**
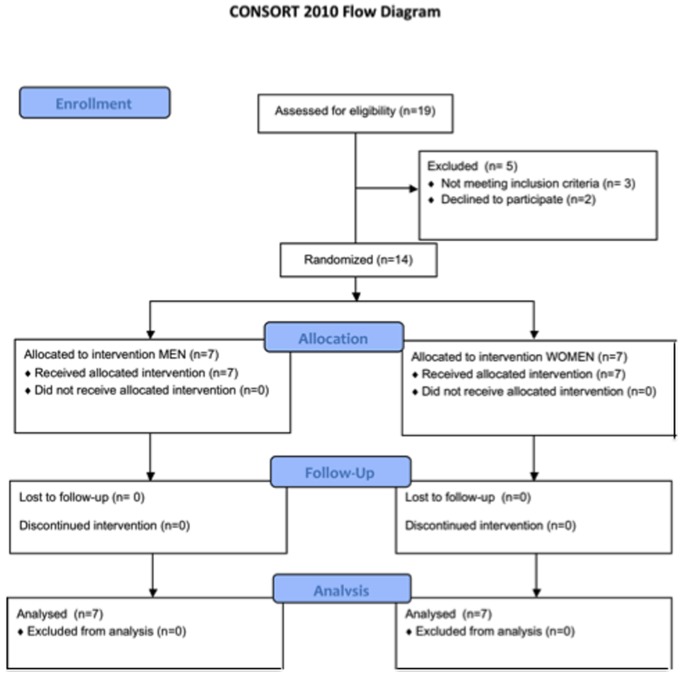
Flow diagram of participants through all phases of the trial.

Resting blood pressure was measured 24 h after the final training session, before subjects were fitted with the CGM and Actiheart. CGM data was collected for a 24 h period starting ∼48 h after the final exercise session and diet was controlled to be the same as baseline. There was no difference in activity counts between the baseline and post-testing CGM period (P>0.05). Fasting blood and a resting muscle biopsy sample were obtained 72 h following the last training bout. Approximately 4 d following the biopsy and 1 wk after the final training session, a maximal exercise test was performed using the same procedures as at baseline. All procedures and controls were identical to those employed during baseline testing and took place during July-September 2013. A flow chart of all participants involved in the trial is depicted in Figure 1.

### Blood Analysis

Plasma glucose was analyzed using a kit assay (Pointe Scientific, Canton MI, USA) and serum insulin was measured by ELISA according to manufacturer instructions (ALPCO Immunoassays, Salem NH, USA). Insulin resistance was calculated using HOMA-IR [18].

### Muscle Analysis

#### Enzyme activity

One piece of muscle (∼25 mg) was homogenized in Lysing Matrix D tubes (MP Biomedicals, Solon, OH, USA) using the FastPrep-24 Tissue and Cell Homogenizer (MP Biomedicals, Solon, OH, USA) for 10×5 s cycles at a speed of 4 m/s with samples placed on ice for 5 min in between cycles. Samples were homogenized in 20 volumes of buffer containing 70 mM sucrose, 220 mM mannitol, 10 mM HEPES supplemented with protease inhibitors (Complete Mini, Roche Applied Science, Laval, PQ, Canada) and used to determine the maximal activity of citrate synthase and 3-β-hydroxyacyl CoA dehydrogenase (β-HAD) as we have previously described [14], [19], [20]. Protein concentration of homogenates was determined using a commercial assay (BCA Protein Assay, Pierce, Rockford, IL, USA) and enzyme activity is expressed as mmol/kg protein/hr.

#### Western blotting

A second piece of muscle (∼30 mg) was homogenized in RIPA buffer using Lysing Matrix D tubes (MP Biomedicals, Solon, OH, USA) with the FastPrep-24 Tissue and Cell Homogenizer (MP Biomedicals, Solon, OH, USA). Samples were processed for 4×20 s cycles at 4.0 m/s, with samples placed on ice for 5 min in between cycles, followed by 2×20 s cycles at 4.0 m/s, with samples placed on ice for 2 min in between cycles. Western blot analysis was conducted using techniques described previously [14], [19]. Briefly, protein concentration of homogenates was determined (BCA Protein Assay) and equal amounts of protein were prepared in 4× Laemmli's buffer and heated to 95°C before being separated by 10% SDS-PAGE and electrotransferred to nitrocellulose membranes. Ponceau S staining was performed following transfer to visualize equal loading and transfer. Following 1 h blocking in 5% fat-free milk Tris-buffered saline 0.1% Tween 20 (TBS-T), membranes were incubated in the primary antibody (glucose transporter 4; Millipore, AB1345 or COXIV; Mitosciences, MS408) overnight at 4°C in 3% fat-free milk TBS-T based on previously optimized conditions. After 3×5 min washes in TBS-T, membranes were incubated in the species-specific secondary antibody diluted (1∶10,000) in 3% fat-free milk TBS-T for 1 h at room temperature, washed in TBS-T for 6×5 min, and visualized by chemiluminescence (SuperSignal West Dura, Pierce) using a FluorChem SP Imaging System (Alpha Innotech Corporation, San Leandro, CA, USA). ImageJ software (NIH) was used to quantify the optical density of protein bands. Protein content was expressed as a fold change relative to pre-training for all subjects. α-tubulin (Cell Signaling Technology, #2125), which did not change following training (p = 0.91), was used as a loading control.

### Statistical Analysis

All data were analyzed using a two-factor analysis of variance (ANOVA), with the between factor group (men, women) and the within factor time (pre-, post-training) using SPSS Statistics software. Significant interactions and main effects were subsequently analyzed using a Tukey's honestly significant difference post hoc test. The level of significance for all analyses was set at P<0.05 and all data are presented as means ± S.D for n = 7 in each group, except for the CGM data which represents n = 6 per group.

## Results

### Descriptive characteristics of training

**Figure 2:**
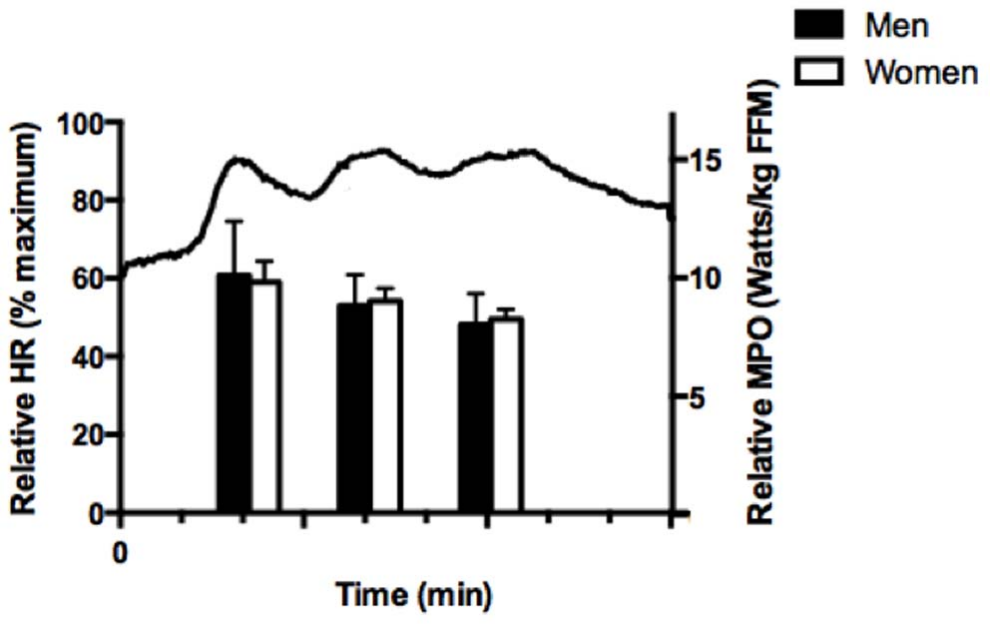
Characterization of the low-volume SIT protocol. Solid line represents average heart rate (HR) response, expressed as a % of maximum, for all subjects during the first training session (left side y-axis). Bar graph represents relative mean power output (MPO) per kilogram fat-free mass (FFM) for men (dark bar) and women (white bar) during the first training session (right side y-axis).

Adherence to the training sessions was 100%. Mean HR, measured continuously during the first training session and averaged over the entire 10 min protocol including warm-up and cool-down, was 83±2% of HR_max_. Relative PPO and MPO measured on the first and last training session did not differ between men and women and increased with training (Table 2, main effect for time, p<0.01). The average HR response for all subjects and average MPO for men and women during session 1 is depicted in Figure 2.

**Table 2:** Markers of Health and Fitness.

	MEN		WOMEN	
VARIABLE	PRE	POST	PRE	POST
Body Mass (kg)	77±12	77±13	79±15	79±15
FPG (mmol/L)	5.1±0.3	5.2±0.3	5.0±0.3	5.0±0.3
FPI (uIU/ml)	13.5±7.9	10.7±7.0*	9.6±4.0	7.1±3.0*
HOMA-IR	3.1±1.9	2.5±1.5*	2.1±0.9	1.5±0.6*
Gmax (mmol/L)	8.0±1.3	6.8±1.1*	7.3±0.6	7.6±0.9
Resting SBP (mmHg)	124±8	116±8*	109±11	100±11*
Resting DBP (mmHg)	71±11	67±5	66±9	60±9
Resting MAP (mmHg)	88±8	83±4*	80±10	74±9*
Relative PPO (W/kg FFM)	11.3±4.1	12.2±3.6*	10.0±0.6	11.8±1.1*
Relative MPO (W/kg FFM)	9.0±1.6	10.6±1.5*	9.0±0.5	12.0±0.1*

Values are means ± S.D. N = 7 for men and women. *Significantly different than pre-training (p≤0.05).

FPG, fasting plasma glucose; FPI, fasting plasma insulin; Gmax: daily peak glucose concentration.

### Skeletal muscle adaptations to very low-volume SIT

**Figure 3:**
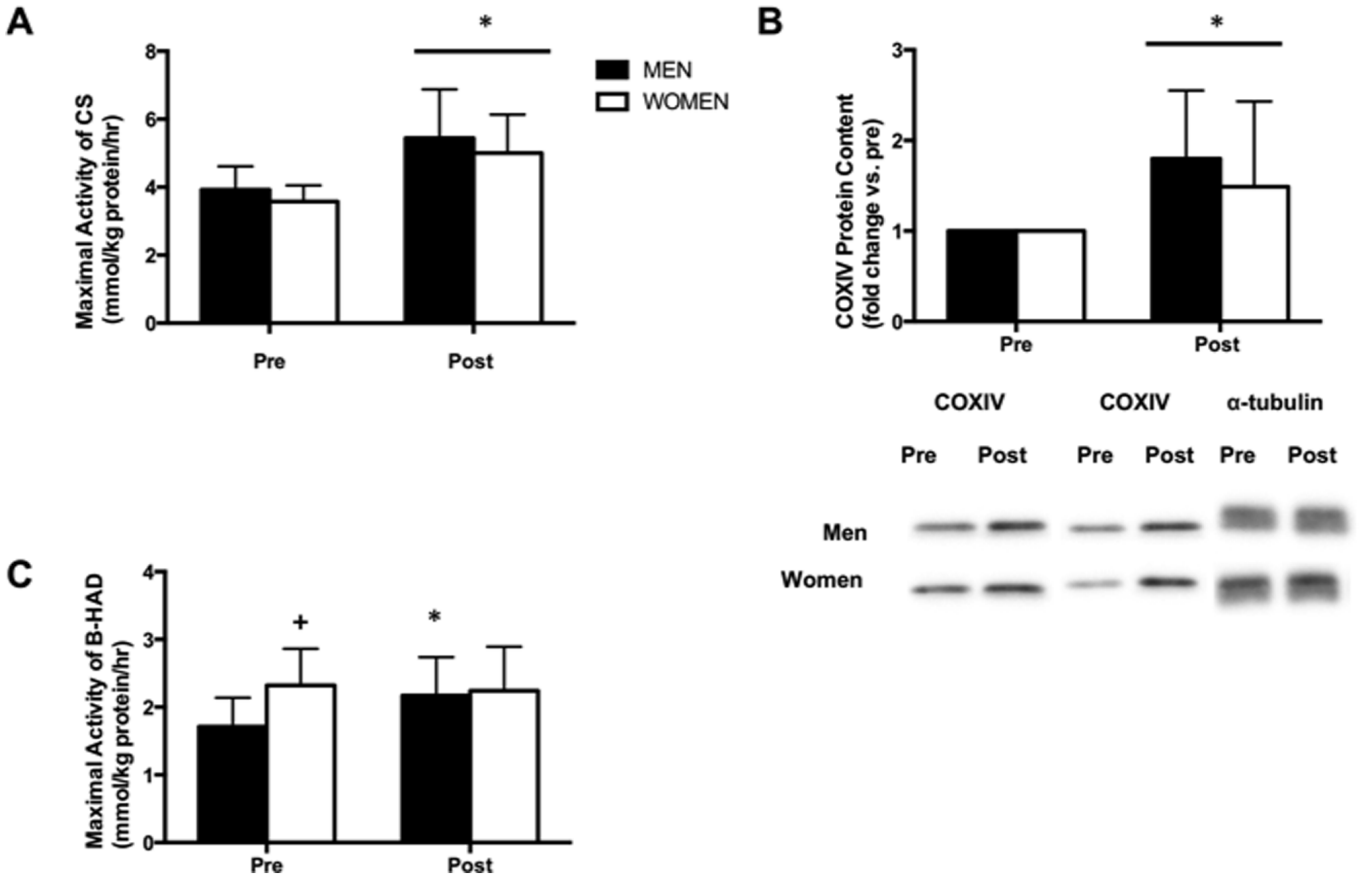
Very low-volume SIT improves skeletal muscle mitochondrial capacity. Measured in muscle biopsy samples obtained from the vastus lateralis before (PRE) and 72 h after (POST) 6-week SIT in men and women. Maximal activity of citrate synthase (A), protein content of COXIV (B) and maximal activity of β-HAD (C). Values are means ± SD (n = 7 per group). Representative Western blots for 2 men and 2 women are shown for COXIV. α-tubulin was used a loading control and representative Western blots are shown. *P<0.05, pre- vs. post-training; +p<0.05, men vs. women at same time point; line denotes a main effect.

**Figure 4:**
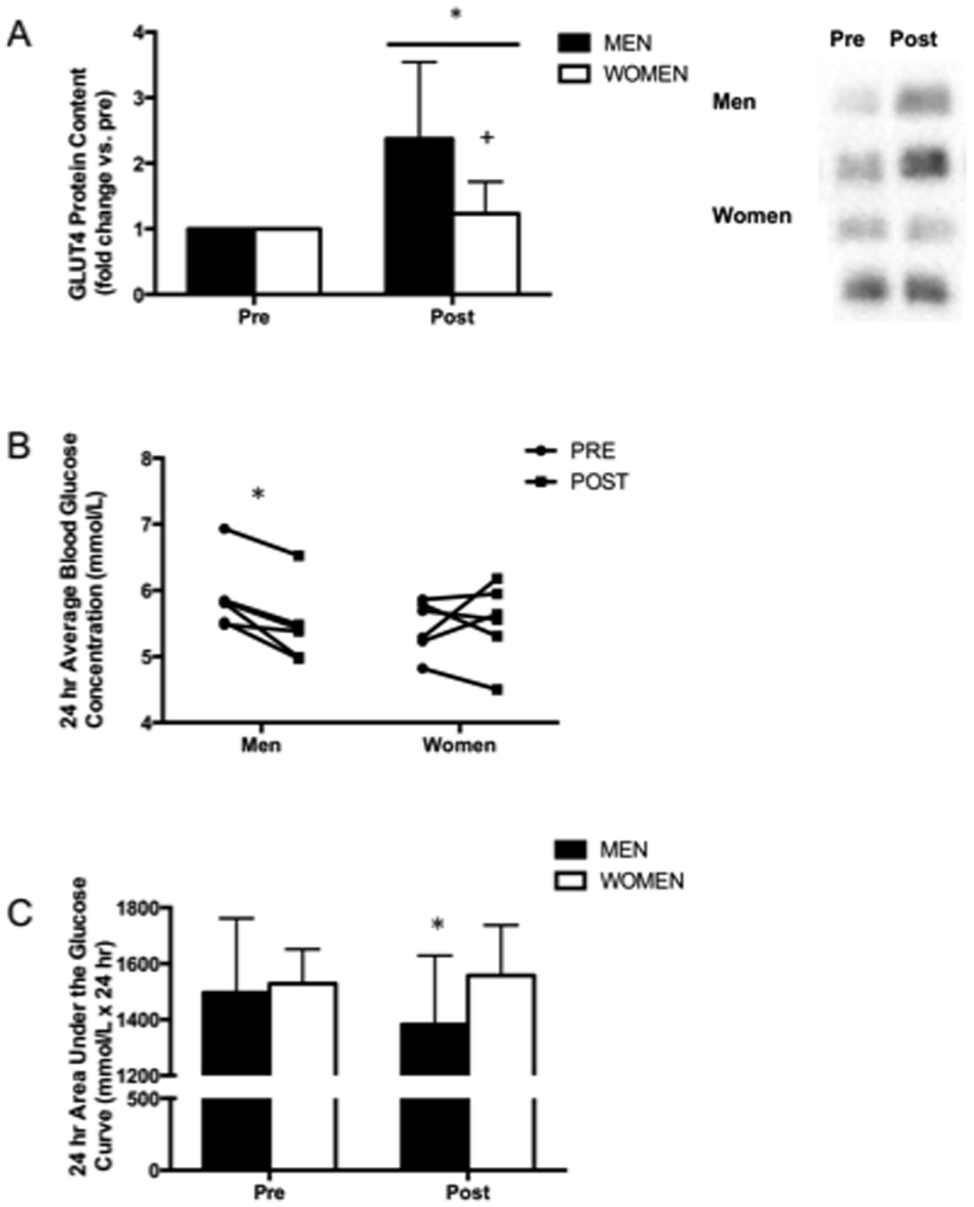
Improved indices of blood glucose control in men following very low-volume SIT. GLUT4 protein content measured in muscle biopsy samples obtained from the vastus lateralis before (PRE) and 72 h after (POST) 6 week SIT in men and women (A). Individual 24 h average blood glucose concentration (B) and 24 h blood glucose area under the curve (AUC) measured before (PRE) and 48–72 h after (POST) 6 week SIT in men and women using continuous glucose monitoring (CGM). Values are means ± SD (n = 7 per group for muscle data, n = 6 per group for CGM data). Representative Western blots for 2 men and 2 women are shown for GLUT4. *P<0.05, pre- vs. post-training; +p<0.05, men vs. women at same time point.

The maximal activity of citrate synthase increased by ∼40% after training (Fig. 3A, main effect for time, p<0.001). COXIV protein content also increased after training with no differences between groups (Fig. 3B, main effect for time, p<0.01), however β-HAD maximal activity only increased after training in the men (Fig. 3C; interaction between training and sex, p<0.05). GLUT4 protein content increased in both men and women following training (Fig. 4A, main effect for time, p<0.01), however men increased to a greater extent compared to women (138% vs. 23%, interaction between training and sex, p<0.05).

### Indices of cardiometabolic health

**Figure 5:**
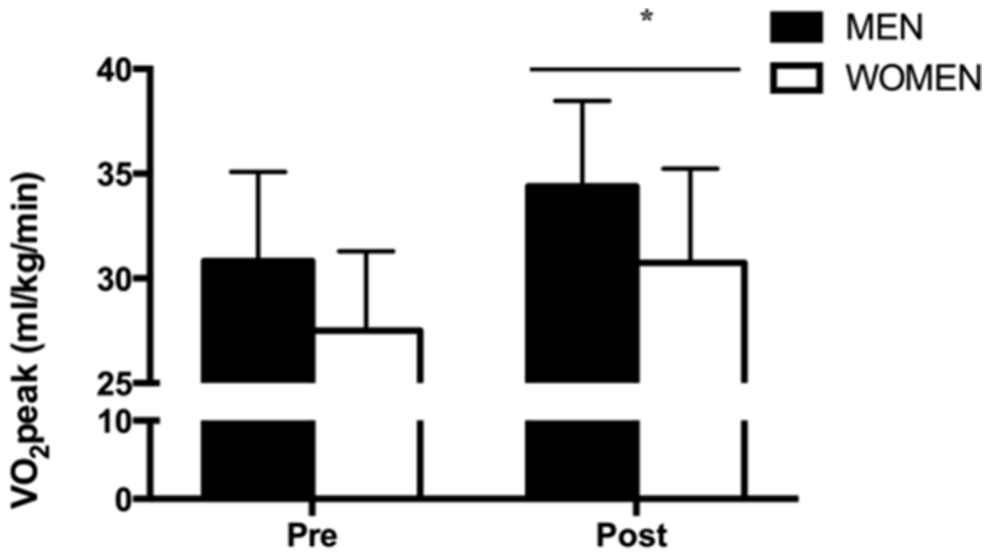
Very low-volume SIT increases VO_2_ peak. Measured before (PRE) and 1 week following (POST) 6 wk SIT in men and women. Values are means ± SD (n = 7 per group). *P<0.05, pre- vs. post-training; line denotes a main effect.

Very low-volume interval training increased VO_2_ peak by 12% in both men and women (Fig. 5, main effect for time, p<0.001), which was associated with a 14% increase in maximal workload (Table 1, main effect for time, p<0.001). Mean arterial pressure (MAP) was reduced by 6% and 8% in men and women respectively following training (Table 2, main effect for time, p<0.01). Systolic blood pressure (SBP) was also reduced following training (Table 2, main effect for time, P<0.01), while diastolic blood pressure (DBP) trended towards being lower (Table 2, p = 0.07). Insulin sensitivity measured by HOMA-IR was improved after training (Table 2, main effect for time, p<0.05), owing mainly to a decrease in fasting serum insulin (Table 2, main effect for time, p≤0.05). There was no change in fasting plasma glucose in either group (Table 2, p>0.05). CGM revealed a lower 24 h average blood glucose concentration after training in men (5.4±0.6 vs. 5.9±0.5 mmol/L, p<0.05) but not women (Fig. 4B, 5.5±0.6 vs. 5.5±0.4 mmol/L, p>0.05). Similarly, 24 h glucose AUC was reduced in men only (Fig. 4C, interaction between training and sex, p<0.05). G_max_ was lower in men following training, but not in women (Table 2, interaction between training and sex, p<0.01)

## Discussion

The main finding from the present study was that short-term interval training, using a protocol that involved only 1 min of very intense exercise within a total time commitment of 10 min, was a potent stimulus to induce physiological adaptations that are linked to improved health in overweight and obese adults. Our general design, which involved 3 sessions per week for 6 wk, was similar to recent studies by Metcalfe [10] and Ma [11], but clarified outstanding questions regarding the potential for very low-volume interval training to increase muscle oxidative capacity, resting blood pressure and aspects of glycemic control. Despite the small sample size, we also found evidence of potential sex-specific adaptations to this type of training that warrant further investigation.

### Very low-volume interval training increases muscle oxidative capacity

A recent systematic review and meta-analysis [21] proposed a classification scheme for interval training in an effort to standardize terminology employed in future studies. Using the descriptors proposed by Weston and colleagues, we have opted to classify the present protocol as “sprint interval training” (SIT) given the “all-out” efforts, as opposed to “high-intensity interval training” (HIIT), which the authors define as bouts performed at relatively intense but nonetheless submaximal workloads corresponding to 80–100% of maximal heart rate [21]. We report here for the first time that very low-volume SIT can increase the maximal activity of citrate synthase, which is reportedly one of the best indicators of mitochondrial content in human skeletal muscle, as it is highly correlated with gold-standard measures made by electron microscopy [22]. While skeletal muscle adaptations to SIT are well established, there are limited and equivocal data regarding the effect of very low-volume SIT on mitochondrial content. Skleryk et al. [23] reported that a protocol involving 8–12×10 s all-out cycle sprints against 5% body weight interspersed with 80 s rest, performed six times over 2 wk, did not improve mitochondrial capacity in overweight men as reflected by a lack of change in the protein content of COXII and COXIV. In contrast, Ma et al. [11] showed that a protocol consisting of 8, 20 s cycling efforts at an intensity of 170% of VO_2_ peak and interspersed with 10 s of recovery, performed four times per week for 4 wk, increased the protein content of COXI and COXIV, however, the maximal activity of citrate synthase was unchanged. The results from the present study confirm that 6 wk of very low-volume SIT, involving a total of only 3 min of all out intermittent exercise within a 30 min time commitment per week, was a sufficient stimulus to elicit a robust increase in citrate synthase similar to what has been reported after protocols involving a larger volume of SIT or traditional moderate-intensity continuous training that involve a much greater total volume of exercise and training time commitment [24]. Clearly, there is some minimal total volume of SIT necessary to acutely stimulate mitochondrial biogenesis, which when performed repeatedly leads to measureable increases in enzyme protein content or maximal activity. The various short-term, very low-volume SIT protocols that have been employed to date are likely on the lower end of this threshold, which may in part explain the equivocal results to date. Additional studies, like the elegant work by Perry et al. [25], which characterized the early time course of adaptation to HIIT, will help to resolve this matter.

### Effect of very low-volume interval training on markers of cardiometabolic health

Seminal work by Tabata and colleagues over two decades ago showed that 7–8 bouts of 20 s all out sprints, with 10 s rest in between, improved VO_2_ peak in young men by 15%, when performed four times per week for 6 wk [26]. The beneficial effect of “Tabata style” training on VO_2_ peak, which is a popular exercise strategy among many personal trainers, was recently confirmed by Ma et al. [11] who reported a 19% increase in young men after 4 wk. The present work, and recent studies by others [10]–[12], [27], confirm that very brief bouts of all-out exercise, performed a few times per week, is a very time efficient strategy to improve VO_2_ peak, which is a strong predictor of all-cause morbidity and mortality [28]. A novel, important finding from the present work was the significant reduction in MAP when measured 24 h after the final training bout, which is of similar magnitude to findings following traditional Wingate-based SIT in overweight/obese men and women [13], as well as 16 wk of high volume aerobic interval or continuous moderate intensity training in individuals with metabolic syndrome [29]. Based on findings from a recent systematic review and meta-analysis, the blood pressure reduction in the present study is of similar magnitude to those following intermittent isometric resistance training [30], which is emerging as a very effective exercise strategy for lowering resting blood pressure [30], [31]. It is unclear if our findings represent an acute effect of the last training bout, however if one performs SIT every other day as in the present study, the beneficial effect on blood pressure would be maintained.

### Potential sex-specific adaptations to low-volume interval training

SIT has been shown to improve insulin sensitivity, based on hyperinsulinemic euglycemic clamps performed on sedentary and recreationally active individuals [2] as well as oral glucose tolerance tests (OGTTs) performed on young healthy [4] and overweight/obese [13] men. Metcalfe et al. [10] recently reported that a 10 min low-intensity cycling protocol that included 2×20 s all-out sprints, performed 18 times over 6 wk, improved insulin sensitivity measured by OGTTs in men but not women [10]. Consistent with the observations of Metcalfe et al. [10], we found using CGM that 24 h average blood glucose concentration, glucose AUC and G_max_, measured under standard dietary conditions from 48–72 h after the final training session, were improved in men but not women. Interestingly, the training-induced increase in total GLUT4 protein content was approximately 6-fold higher in men compared to women.

The lack of change in peripheral glucose control in women in the present study is consistent with recent reports by others [10], [15], although this is not a universal finding [2]. It is possible that our low sample size (n = 6 for CGM data), or the fact that the women had higher 24 h blood glucose control at baseline, influenced our findings and resulted in a type 2 statistical error. Nonetheless, by way of a possible related mechanism, it has also been speculated that the rapid improvement in insulin sensitivity following SIT is attributed to high rates of glycogen breakdown and subsequent re-synthesis following each exercise bout [10], and women have been shown to break down 42% less muscle glycogen in type I fibers during a single Wingate sprint [32]. Future studies are needed to investigate if GLUT4 translocation following acute SIT is blunted in women, and definitively determine in larger cohorts of subjects if improvements in glucose control following SIT are sex-specific. HOMA-IR was improved in both men and women after training, owing to significant reductions in fasting plasma insulin, and consistent with previous studies [3], [33].

We also found sex-specific differences in a marker of lipid oxidation capacity, based on changes in the maximal activity of β-HAD which were detected in men but not women. A similar period of Wingate-based SIT was reported to improve the maximal activity of β-HAD in both men and women, but that study did not involve a specific comparison between sexes [24]. Similar to the pre-training CGM data, it is possible that the higher baseline value for β-HAD in women in the present study reduced their potential to increase the capacity for lipid oxidation compared to men. Other recent studies however, have also highlighted sex-based differences in the skeletal muscle adaptive response to SIT in active young men and women [34]. Scalzo et al. [34] reported higher rates of muscle protein synthesis in men compared to women following a 3 wk SIT intervention, based on oral administration of deuterium oxide. Future research is needed, using designs which control for menstrual cycle phase and initial fitness level [35], to evaluate if sex-based differences exist in the skeletal muscle adaptive response low-volume SIT.

## Conclusions

In summary, we report that 3 min of all-out exercise performed within a 30 min time commitment per week including warm-up and cool-down, improved skeletal muscle oxidative capacity and indices of cardiometabolic health including VO_2_ peak and blood pressure, in overweight/obese adults. The protocol employed in the present study involved a training time commitment that was considerably lower than in previous Wingate-based SIT studies (i.e., 10 versus ∼25 min per session) and provides further evidence of the potential for very brief, intense bursts of exercise to elicit physiological adaptations that are associated with improved health status in a time-efficient manner. Despite the small sample size, potential sex-specific adaptations were apparent that warrant further investigation.

## Supporting Information

Checklist S1 **TREND checklist.**
(PDF)

Protocol S1 **Trial study protocol.**
(DOCX)

## References

[1] Gibala MJ, McGee SL (2008) Metabolic adaptations to short-term high-intensity interval training: a little pain for a lot of gain? Exerc Sport Sci Rev 36: 58–63 10.1097/JES.0b013e318168ec1f 18362686

[2] Richards JC, Johnson TK, Kuzma JN, Lonac MC, Schweder MM, et al (2010) Short-term sprint interval training increases insulin sensitivity in healthy adults but does not affect the thermogenic response to beta-adrenergic stimulation. J Physiol 588: 2961–2972 10.1113/jphysiol.2010.189886 20547683 PMC2956910

[3] Hood MS, Little JP, Tarnopolsky MA, Myslik F, Gibala MJ (2011) Low-Volume Interval Training Improves Muscle Oxidative Capacity in Sedentary Adults. Med Sci Sports Exerc 43: 1849–1856 10.1249/MSS.0b013e3182199834 21448086

[4] Babraj JA, Vollaard NBJ, Keast C, Guppy FM, Cottrell G, et al (2009) Extremely short duration high intensity interval training substantially improves insulin action in young healthy males. BMC Endocr Disord 9: 3 10.1186/1472-6823-9-3 19175906 PMC2640399

[5] Little JP, Gillen JB, Percival M, Safdar A, Tarnopolsky MA, et al (2011) Low-volume high-intensity interval training reduces hyperglycemia and increases muscle mitochondrial capacity in patients with type 2 diabetes. J Appl Physiol 111: 1554–1560 10.1152/japplphysiol.00921.2011 21868679

[6] Trost SG, Owen N, Bauman AE, Sallis JF, Brown W (2002) Correlates of adults' participation in physical activity: review and update. Med Sci Sports Exerc 34: 1996–2001 10.1249/01.MSS.0000038974.76900.92 12471307

[7] Gillen JB, Gibala MJ (2014) Is high-intensity interval training a time-efficient exercise strategy to improve health and fitness? Appl Physiol Nutr Metab 39: 409–412 10.1139/apnm-2013-0187 24552392

[8] Tremblay MS, Warburton DER, Janssen I, Paterson DH, Latimer AE, et al (2011) New Canadian Physical Activity Guidelines. Appl Physiol Nutr Metab 36: 36–46 10.1139/H11-009 21326376

[9] Garber CE, Blissmer B, Deschenes MR, Franklin BA, Lamonte MJ, et al (2011) American College of Sports Medicine position stand. Quantity and quality of exercise for developing and maintaining cardiorespiratory, musculoskeletal, and neuromotor fitness in apparently healthy adults: guidance for prescribing exercise. Med Sci Sports Exerc 43: 1334–1359 10.1249/MSS.0b013e318213fefb 21694556

[10] Metcalfe RS, Babraj JA, Fawkner SG, Vollaard NBJ (2011) Towards the minimal amount of exercise for improving metabolic health: beneficial effects of reduced-exertion high-intensity interval training. Eur J Appl Physiol 112: 2767–2775 10.1007/s00421-011-2254-z 22124524

[11] Ma JK, Scribbans TD, Edgett BA, Boyd JC, Simpson CA, et al (2013) Extremely low-volume, high-intensity interval training improves exercise capacity and increases mitochondrial protein content in human skeletal muscle. J Mol Integr Physiol 3: 202–210.

[12] Hazell TJ, Macpherson REK, Gravelle BMR, Lemon PWR (2010) 10 or 30-S Sprint Interval Training Bouts Enhance Both Aerobic and Anaerobic Performance. Eur J Appl Physiol 110: 153–160 10.1007/s00421-010-1474-y 20424855

[13] Whyte LJ, Gill JMR, Cathcart AJ (2010) Effect of 2 weeks of sprint interval training on health-related outcomes in sedentary overweight/obese men. Metabolism 59: 1421–1428 10.1016/j.metabol.2010.01.002 20153487

[14] Little JP, Safdar A, Wilkin GP, Tarnopolsky MA, Gibala MJ (2010) A practical model of low-volume high-intensity interval training induces mitochondrial biogenesis in human skeletal muscle: potential mechanisms. J Physiol 588: 1011–1022 10.1113/jphysiol.2009.181743 20100740 PMC2849965

[15] Gillen JB, Percival ME, Ludzki A, Tarnopolsky MA, Gibala MJ (2013) Interval training in the fed or fasted state improves body composition and muscle oxidative capacity in overweight women. Obesity. doi:10.1002/oby.20379. 10.1002/oby.20379 23723099

[16] Frankenfield D, Roth-Yousey L, Compher C (2005) Comparison of predictive equations for resting metabolic rate in healthy nonobese and obese adults: a systematic review. J Am Diet Assoc 105: 775–789 10.1016/j.jada.2005.02.005 15883556

[17] Tarnopolsky MA, Pearce E, Smith K, Lach B (2011) Suction-modified Bergström muscle biopsy technique: experience with 13,500 procedures. Muscle Nerve 43: 717–725 10.1002/mus.21945 21462204

[18] Matsuda M, DeFronzo RA (1999) Insulin sensitivity indices obtained from oral glucose tolerance testing. Diabetes Care 22: 1462–1470. 10480510 10.2337/diacare.22.9.1462

[19] Gibala MJ, Little JP, van Essen M, Wilkin GP, Burgomaster KA, et al (2006) Short-term sprint interval versus traditional endurance training: similar initial adaptations in human skeletal muscle and exercise performance. J Physiol 575: 901–911 10.1113/jphysiol.2006.112094 16825308 PMC1995688

[20] Carter SL, Rennie CD, Hamilton SJ, Tarnopolsky MA (2001) Changes in skeletal muscle in males and females following endurance training. Can J Physiol Pharmacol 79: 386–392 10.1139/cjpp-79-5-386 11405241

[21] Weston KS, Wisløff U, Coombes JS (2013) High-intensity interval training in patients with lifestyle-induced cardiometabolic disease: a systematic review and meta-analysis. Br J Sports Med. doi:10.1136/bjsports-2013-092576. 10.1136/bjsports-2013-092576 24144531

[22] Larsen S, Nielsen J, Hansen CN, Nielsen LB, Wibrand F, et al (2012) Biomarkers of mitochondrial content in skeletal muscle of healthy young human subjects. J Physiol 590: 3349–3360 10.1113/jphysiol.2012.230185 22586215 PMC3459047

[23] Skleryk JR, Karagounis LG, Hawley JA, Sharman MJ, Laursen PB, et al (2013) Two weeks of reduced-volume sprint interval or traditional exercise training does not improve metabolic functioning in sedentary obese men. Diabetes Obes Metab 15: 1146–1153 10.1111/dom.12150 23802920

[24] Burgomaster KA, Howarth KR, Phillips SM, Rakobowchuk M, Macdonald MJ, et al (2008) Similar metabolic adaptations during exercise after low volume sprint interval and traditional endurance training in humans. J Physiol 586: 151–160 10.1113/jphysiol.2007.142109 17991697 PMC2375551

[25] Perry CGR, Lally J, Holloway GP, Heigenhauser GJF, Bonen A, et al (2010) Repeated transient mRNA bursts precede increases in transcriptional and mitochondrial proteins during training in human skeletal muscle. J Physiol 588: 4795–4810 10.1113/jphysiol.2010.199448 20921196 PMC3010147

[26] Tabata I, Nishimura K, Kouzaki M, Hirai Y, Ogita F, et al (1996) Effects of moderate-intensity endurance and high-intensity intermittent training on anaerobic capacity and VO2max. Med Sci Sports Exerc 28: 1327–1330. 8897392 10.1097/00005768-199610000-00018

[27] Tjønna AE, Leinan IM, Bartnes AT, Jenssen BM, Gibala MJ, et al (2013) Low- and high-volume of intensive endurance training significantly improves maximal oxygen uptake after 10-weeks of training in healthy men. PLoS One 8: e65382 10.1371/journal.pone.0065382 23734250 PMC3667025

[28] Blair SN, Brodney S (1999) Effects of physical inactivity and obesity on morbidity and mortality: current evidence and research issues. Med Sci Sports Exerc 31: S646–62. 10593541 10.1097/00005768-199911001-00025

[29] Tjønna AE, Lee SJ, Rognmo Ø, Stølen TO, Bye A, et al (2008) Aerobic interval training versus continuous moderate exercise as a treatment for the metabolic syndrome: a pilot study. Circulation 118: 346–354 10.1161/CIRCULATIONAHA.108.772822 18606913 PMC2777731

[30] Cornelissen VA, Smart NA (2013) Exercise training for blood pressure: a systematic review and meta-analysis. J Am Heart Assoc 2: e004473 10.1161/JAHA.112.004473 23525435 PMC3603230

[31] Brook RD, Appel LJ, Rubenfire M, Ogedegbe G, Bisognano JD, et al (2013) Beyond medications and diet: alternative approaches to lowering blood pressure: a scientific statement from the american heart association. Hypertension 61: 1360–1383 10.1161/HYP.0b013e318293645f 23608661

[32] Esbjörnsson-Liljedahl M, Sundberg CJ, Norman B, Jansson E (1999) Metabolic response in type I and type II muscle fibers during a 30-s cycle sprint in men and women. J Appl Physiol 87: 1326–1332. 10517759 10.1152/jappl.1999.87.4.1326

[33] Trapp E, Heydari M, Freund J, Boutcher SH (2008) The effects of high-intensity intermittent exercise training on fat loss and fasting insulin levels of young women. Int J Obes 32: 684–691 10.1038/sj.ijo.0803781 18197184

[34] Scalzo RL, Peltonen GL, Binns SE, Shankaran M, Giordano GR, et al (2014) Greater muscle protein synthesis and mitochondrial biogenesis in males compared with females during sprint interval training. FASEB J 28: 1–10 10.1096/fj.13-246595 24599968

[35] Tarnopolsky MA (2008) Sex differences in exercise metabolism and the role of 17-beta estradiol. Med Sci Sports Exerc 40: 648–654 10.1249/MSS.0b013e31816212ff 18317381

