# Supplementary material for: Three Minutes of All-Out Intermittent Exercise per Week Increases Skeletal Muscle Oxidative Capacity and Improves Cardiometabolic Health
Source: PLoS One. 2014 Nov 3;9(11):e111489. doi: 10.1371/journal.pone.0111489 (PMC4218754; doi:10.1371/journal.pone.0111489)
Supplement: Protocol S1 — Trial study protocol. (DOCX) [file pone.0111489.s002.docx]

**RESEARCH PROTOCOL**

Project Title

Effect of very low volume exercise training on skeletal muscle remodeling

Principle Investigator

Martin Gibala, Ph.D.

Professor and Chair

Department of Kinesiology

McMaster University

Co-Investigator

Mark Tarnopolsky, M.D., Ph.D.

Professor

Departments of Pediatrics and Medicine

McMaster University

Student Co-Investigator

Jenna Gillen, B.Sc

Ph.D. Student

Department of Kinesiology

McMaster University

**INTRODUCTION**

Current lifestyle trends characterized by inactivity and poor dietary habits have led to a dramatic decline in the health of Canadians. In fact, among men and women aged 15-69, 47% have poor aerobic fitness and 60% are classified as overweight or obese (1,2). The health consequences of excess weight are well known and include among others, an increased risk for the development of cardiovascular disease and type 2 diabetes (3). A common precursor to these metabolic disorders is peripheral insulin resistance (4). While the metabolic causes of insulin resistance are widespread and complex, there is increasing evidence that physical inactivity could be the major initiating factor (5). On the contrary, regular exercise is an effective strategy to combat insulin resistance while also improving aerobic fitness, body composition and skeletal muscle metabolic health (3). Despite this knowledge and public health guidelines recommending that adults accumulate at least 150 minutes of moderate to vigorous intensity activity each week (3), 85% of Canadian’s do not meet these minimum physical activity guidelines (6). The potential to establish more practical time-efficient exercise strategies that improve skeletal muscle metabolic health and reduce the risk of chronic disease has enormous public health and health care spending implications.

Reasons for not engaging in regular exercise are numerous and complex, however a “lack of time” continues to be cited as a key barrier (7). Physical activity guidelines like those from Health Canada typically focus on a minimum number of minutes per week. An increasing body of evidence, however, suggests that intensity of exercise performed, and not necessarily the duration, is important when attempting to improve the health and fitness of the population. A recent 18 yr observational study which tracked activity patterns of over 5000 participants found that the relative intensity and not duration of cycling was related to all-cause mortality (8). Regardless of the cycling duration (even when under 30 min/wk), higher relative intensity was associated with much lower mortality rates. Similarly, Rosenkilde et al. (9) found that a group of overweight men who exercised for 30 min per day over 13 wks lost the same amount of fat mass as a group who trained 60 min per day. Based off of these findings, it appears that focusing on the duration of exercise may not be necessary for improving overall health. Perhaps developing short-duration, high-intensity exercise protocols would be an effective as well as appealing strategy to improve the fitness of the population.

High-intensity interval training, which consists of short bursts of intense exercise separated by periods of recovery, has repeatedly been shown to induce numerous performance, metabolic and health adaptations that resemble high-volume endurance training (10). Work from our laboratory over the past few years has specifically focused on a practical, time-efficient HIT model which consists of 10 x 1 minute cycle sprints at 90% maximal heart rate with 1 minute of recovery in between, totalling 20 min per session. We have shown that 2-6 weeks of low-volume HIT increases skeletal muscle mitochondrial capacity, glucose transporter (GLUT) 4 protein content and maximal oxygen uptake to the same extent as endurance training, despite a dramatically reduced time commitment associated with HIT (11,12). Additionally, this form of training has been shown to improve insulin sensitivity in previously sedentary adults (12) and glycemic control in patients with type 2 diabetes (13). We have also found 6 wk of HIT reduces fat mass and increases lean mass in overweight women (Gillen et al., unpublished observations) which is consistent with findings from Trapp et al. suggesting 15 wk of low-volume HIT is superior to traditional endurance-type training for reducing total body mass and abdominal adiposity in young women (14). The 10 x 1min HIT protocol involves only 30 min of exercise within a 75 min time commitment per week including warm up and cool down, which equates to half that recommended by public health guidelines. This appears to highlight the efficacy of exercise intensity rather than duration in mediating physiological adaptations to training.

Two recent studies investigated the effect of extremely low-volume HIT models on markers of cardiorespiratory fitness and health. Metcalfe et al.(15) showed that as little as 2 20 s sprints during 10 min of low-intensity cycling 3x/wk improved VO_2_peak and insulin sensitivity (based on oral glucose tolerance tests) in young healthy males after only 2 wk. Similarly, Jakeman et al. (16) showed that 6 sessions of 10 x 6 s sprints with 1 min of recovery improved 10 km time trial performance. These preliminary studies are promising and appear to suggest that a 30 min time commitment per week could be sufficient to improve health and aerobic fitness if the exercise is performed at a high intensity. Additional studies are warranted with more comprehensive analyses including skeletal muscle adaptations to these very low-volume HIT protocols. Examining changes in skeletal muscle oxidative and substrate transport capacity, body composition and glycemic control in overweight individuals following 6 wk of very low-volume HIT will shed light on the value of exercise intensity in the face of a very low total time commitment. Therefore we aim to investigate the effectiveness of a HIT model requiring a 10 minute time commitment per day, 3 times per week, in an attempt to identify a practical and a time-efficient exercise strategy which can improve skeletal muscle metabolic capacity and glycemic control in overweight individuals.

**METHODS**

**Subjects**

Twenty men and women (n=10 each) between the ages of 18-45 years will be recruited through poster advertisement.

Inclusion criteria will include:

- Classification of “sedentary” based off a International Physical Activity Questionnaire (IPAQ) score of < 600 MET min/wk
- Body mass index (BMI) > 25/27 < 30kg/m

Exclusion criteria will include:

- Active cardiovascular disease
- Cerebrovascular disease including previous stroke or aneurysm (large vessel or intracranial
- Respiratory disease including pulmonary hypertension of COPD
- Metabolic disease including: parathyroidism, hyper/hypothyroidism, Cushing’s disease or type 1 or 2 diabetes
- Active inflammatory bowel disease
- Renal disease
- Malignancy
- Recent steroid treatment (within 6 mo), or hormone replacement therapy
- Clotting dysfunction
- Musculoskeletal or neurological disorders
- Any disease requiring long-term drug prescriptions, including statins

Details of the experimental protocol, purpose and potential risks of participation will be explained to all subjects and written informed consent will be obtained. Sample size is based on estimated changes in primary outcome variables and standard deviations obtained from analysis of previous studies. 8 subjects are required to detect changes at an alpha level of 0.05 with 80% power. To preserve power we will recruit 10 males and 10 females.

**Overview of Experimental Protocol**

This study consists of 4 phases: i) An initial screening visit; ii) Baseline Testing iii) 6wk of HIT; and iv) Post-training Testing; All phases are outlined below, with additional information on selected procedures following. Figure 1 at the end of the document depicts the experimental protocol.

**I) Initial Screening Visit**

Before subjects formally enter the study, they will be asked to report to the laboratory to ensure they are eligible for participation. During this visit (approximately 45 minutes), subjects will i) read and sign a consent form for the study; ii) conduct a health assessment (questionnaire, height, weight, resting heart rate and blood pressure); iv) provide a fasting blood sample obtained by venupuncture (8ml) and v) complete a physical activity questionnaire.

**II) Baseline Testing**

Once we determine that subjects have met our inclusion criteria, we will invite them to enter the baseline testing phase of the study. This period will include 4 visits to the laboratory: 1) Insertion of Continuous Glucose Monitor (CGM) and accelerometer (Actiheart) which will be worn for 72 hr; 2) DEXA and skeletal muscle biopsy; 3) A VO_2peak_ test; 4) HIT familiarization to become acquainted with the exercise protocol. Subjects will also be asked to complete at 3 d diet record.

**IV) Exercise Training (HIT)**

The exercise training will be 100% supervised and will consist of 3 sessions per week for 6 weeks. All sessions will begin with a 2 minute warm up followed by 6x20 second cycle sprints against 7.5% of their body weight interspersed with 1 minute of recovery. Heart rate and rating of perceived exertion will be monitored at each session. On the first training session participants will be asked to wear the CGM and Actiheart during exercise and for the following 48 hr to identify the acute effect of HIT on glycemic control.

**V) Post-training Testing**

24-48 hrs following the last training bout we will measure resting blood pressure and HR and insert the CGM and Actiheart which will be worn for the following 48 hr (time of interest will be 48-72 hr post training). 72 hr following the last training session participants will undergo a DEXA scan, skeletal muscle biopsy and fasting blood sample. We will also obtain the CGM and Actiheart at this time. At least 72 hr following the biopsy, participants will report to the laboratory for a VO_2peak_ test.

**PROCEDURES**

**Resting Blood Pressure and Heart Rate Measures**

An automated device will be used for measurement of resting HR and BP. Participants will be instructed to rest for 10 minutes prior to the measurement. HR and BP will be measured twice from the dominant arm while the subject is in supine position. This will occur at the initial screening visit and 24 hr post training.

**Fasting Blood Sample**

Following a 10 hr overnight fast on the initial screening visit and 72 hr post training, we will obtain a fasting plasma and serum sample by venupuncture (8ml).

**Continuous Glucose Monitoring (CGM)**

In order to assess the effects of i) a single HIT session and ii) 6 wk of HIT on glycemic regulation under free-living conditions, continuous glucose monitoring (CGM) will be employed for a control day during baseline testing (72 hr), during and after the first HIT session (72 hr) and during post-training testing (48 hr). CGM involves inserting a tiny sensor into the subcutaneous tissue of the abdomen which is connected to a continuous glucose measuring device (CGMS iPro, Medtronic, Northridge, CA). In order to standardize the quantity and timing of nutritional intake, subjects will be provided with individualized, standardized diets to consume during the 72 hr monitoring period. Food intake logs will be utilized to ensure compliance with the standardized diet and to verify meal timing. We have employed this technique in our laboratory previously in patients with type 2 diabetes (13,17)

**Actiheart**

In order to monitor physical activity levels, participants will wear the Actiheart on 3 occasions while wearing the CGM: for 72hr during baseline testing, 72 hr during training and 48hr during post-training testing. The Actiheart is a compact, chest-worn monitoring device that records heart rate and physical activity. It is non-invasive, weighs less than 10 grams and is waterproof. The Actiheart has two clips which attach directly to standard ECG electrodes. One electrode is adhered at V1 or V2 (4^th^ intercostals) and the second electrode is placed approximately 10 cm to the left at V4 or V5. This placement can be adjusted to be comfortable for the subject (i.e. can be positioned above or below the breasts for females). The number of R-waves detected is recorded in 15, 30 or 60 second epochs. Simultaneously, an internal accelerometer senses the frequency and intensity of the subject’s torso movements. Energy expenditure is calculated from both the HR and activity data.

**Dual X-Ray Absorptiometry (DEXA) Scan**

Body composition will be measured by DEXA (Lunar Prodigy Advance, Madison, WI, USA) before and after training. This is a painless non-invasive procedure that uses a small amount of radiation to assess how much fat, bone, and lean tissue is in the entire body. One trained technician will conduct all scans.

**Skeletal Muscle Biopsy Procedure**

Using standard procedures, muscle biopsy samples will be obtained under local anaesthesia (2% lidocaine) from the *vastus lateralis* several days before, at 6 wk and after training using a Bergstom needle adapted with suction (18). The obtained biopsy samples (~100-150 mg each, wet weight) are immediately dissected from any visible fat or connective tissue, sectioned into several pieces, frozen in liquid nitrogen, and stored at -80ºC until subsequent analysis. For immunohistochemistry, a small piece of muscle (approximately 20mg) will be oriented in cross section, mounted in Optimum Cutting Temperature (OCT) compound (Tissue-Tek, Sakura Finetek, USA) and frozen in isopentane cooled with liquid nitrogen. The mounted samples will be stored at -80°C pending analysis.

**VO_2_peak Test**

Subjects will complete an incremental maximal oxygen uptake (VO_2_max) test on a cycle ergometer (Lode Excalibur Sport V 2.0, Groningen, The Netherlands). A metabolic cart with an on-line gas collection system (Moxus modular oxygen uptake system, AEI Technologies, Pittsburgh, PA) will acquire oxygen consumption (VO_2_) and carbon dioxide production (VCO_2_) data and heart rate will be monitored continuously with a heart rate monitor (Polar A3, Lake Success, NY). Following a 5 min warm-up at 50 watts (W) the resistance will be increased by 1 W every two seconds until volitional exhaustion or the point at which pedal cadence falls below 40 rpm. Heart rate will be monitored continuously throughout the test via telemetry with a heart rate monitor (Polar A3, Lake Success, NY). VO_2peak_ and maximal workload (Wmax) will be defined as the highest oxygen consumption and power output values achieved over a 30 s period. The subject is deemed to have reached VO_2_max if: i) the perceived exertion is >19 (Borg Scale); ii) their HR ± 5bpm of age-predicted maximal HR; iii) their RER is >1.2; and iv) a plateau has been reached in their oxygen consumption.

**Dietary and Physical Activity Controls**

Participants will be instructed to make no changes to their diet over the course of the training intervention, which will be verified by 3 d food records before and after training. Additionally, subjects will be asked to refrain from initiating any other form of exercise into their daily routine.

**DATA ANALYSIS**

Fasting Blood Sample

Whole blood will immediately be used for determination of fasting blood glucose concentration using a YSI Glucose and Lactate Analyzer. The remaining plasma and serum samples will be separated by centrifugation, aliquoted and stored at -20°C until analyzed for insulin (commercial ELISA kit) and free fatty acids (colorimetric assay).

Continuous Glucose Monitor (CGM)

The CGM gives us a continuous reading of blood glucose concentration for the duration in which it is worn. We will calculate values such as area under the curve, average glucose concentration and the postprandial glucose response as we have described previously (13,17).

Muscle Biopsies

Mitochondrial Enzyme Activity: Mitochondrial capacity will be assessed by measuring the maximal activity of citrate synthase (CS), cytochrome *c* oxidase (COX) and β-hydroxyacyl-CoA dehydrogenase (β-HAD). Briefly, skeletal muscle biopsy samples (~20 mg) will be homogenized using a glass-on-glass homogenizer in 25 volumes of homogenization buffer (70 mM sucrose, 220 mM mannitol, 10mM HEPES) supplemented with protease inhibitors (Complete Mini^®^, Roche Applied Science, Laval, PQ, Canada). The lysate will be centrifuged at 700 *g* for 10 min at 4°C to pellet cellular debris. Citrate synthase activity will be determined by measuring the formation of thionitrobenzoate anion, as previously described (19). Absorbance is recorded at 412 nm every 30 s for 3 min at 37°C. Mitochondrial electron transport chain complex IV (cytochrome *c* oxidase) activity will be determined by measuring the rate of oxidation of fully reduced cytochrome *c*, as previously described by our group (19). Absorbance is recorded at 550 nm every 30 s for 3 min at 37 °C. The maximal activity of β-hydroxyacyl-CoA dehydrogenase (β-HAD) will be measured using the methods described by Chi et al. (11). All samples will be analyzed in duplicate on a spectrophotometer (Cary Bio-300, Varion, Inc., Palo Alto, CA). Protein concentration of homogenates will be determined using a commercial assay (BCA Protein Assay, Pierce, Rockford, IL, USA).

Western Blotting: Whole muscle lysates for immunoblotting will be prepared by homogenizing ~30 mg of wet muscle in 0.5 mL of RIPA buffer (50 mM HCL, 150 mM NaCl, 2 mM EDTA, 1% NP-40, and 0.1% SDS supplemented with protease and phosphatase inhibitors) using an electronic homogenizer followed by sonication. Homogenates are centrifuged at 15 000 g for 10 min at 4°C and the supernatant is analyzed as the whole muscle fraction. Protein concentration is determined using the BCA method (Pierce, Rockford, IL, USA). The protein content of mitochondrial enzymes (CS, COX subunit II, COX subunit IV, PDH, CPT) as well as glucose and fatty acid transporters (GLUT1, GLUT4, FAT/CD36, FATpm) will be measured using standard Western blotting procedures as previously described (20). Briefly, equal amounts of protein are loaded onto SDS-PAGE gels and separated by electrophoresis for 2-2.5 hours at 100V. Proteins are wet transferred to nitrocellulose membranes for 1 hr at 100V. Ponceau S staining is performed following the transfer to control for equal loading and transfer between lanes. Membranes are blocked at room temperature (RT) by incubating in 5% fat-free milk TBS-T. Blots are then incubated with primary antibodies overnight at 4 °C in 3% fat-free milk or BSA TBS-T. After incubation in appropriate secondary antibody for 1 hour at RT, proteins are detected by chemiluminescence (Supersignal® West Dura, Pierce) and quantified by spot densitometry using Fluorochem® SP Imaging system and software (Alpha Innotech Corporation, San Leandro, CA, USA).

Immunohistochemistry: Muscle cross-sections (7 µm) will be stained with antibodies against A4.951 [myosin heavy chain type I (MHCI; slow isoform); neat; DSHB]; myosin heavy chain type II (MHCII; fast isoform; 1:1000; ab91506, Abcam, Cambridge, MA, USA); laminin (1:1000; ab11575, Abcam, Cambridge, MA, USA); COX subunit IV (COXIV; 1:400; MS408, Mitosciences, Cambridge, MA, USA); and CD31. This analysis will allow us to measure fibre specific capillarization and mitochondrial capacity before and after training. Sections will be fixed in 4% paraformaldehyde (PFA, Sigma, USA) for 10 minutes followed by several washes in phosphate buffered saline with 2% Tween® 20 (PBS-T). Sections will then be incubated for 90 minutes in a blocking solution containing 1% BSA and 5% goat serum. Following blocking, sections will be incubated with the appropriate primary antibody overnight at 4ºC. After several washes in PBS-T sections will be incubated with the appropriate secondary antibody for 120 minutes at room temperature. Sections will then be washed with PBS and 4’,6-diamidino-2-phenylindole (DAPI) (Sigma, USA) for nuclear staining prior to cover slipping with fluorescent mounting media (DAKO, Burlington, ON, Canada). Images will be acquired with a Nikon Eclipse Ti microscope at 20x magnification and captured with a Photometrics CoolSNAP HQ^2^ fluorescent camera (Nikon Instruments, Melville, NY, USA).

**STATISTICAL ANALYSIS**

All data will be analyzed using a paired Student’s t-test with significance set at p ≤ 0.05.

**PERSPECTIVES AND SIGNIFICANCE**

The proposed study will add to the growing body of literature suggesting low-volume HIT is a highly potent and time efficient exercise strategy for improving metabolic health. This will be the first study to assess changes in skeletal muscle oxidative capacity, glycemic control and body composition in overweight individuals following a HIT protocol involving a total time commitment of less than 30 minutes per week. Considering the most commonly cited barrier to regular exercise is a “lack of time” identifying short-duration, high-intensity exercise protocols could be an effective and appealing strategy to improve fitness of the population.

Figure 1. Overview of Experimental Protocol

**References:**

1. Statistics Canada. Health Fact Sheet Aerobic fitness of Canadians, 2009 to 2011. 2012.

2. Statistics Canada. Health Fact Sheet Body composition of Canadian adults, 2009 to 2011. 2012.

3. Tremblay MS, Warburton DER, Janssen I, Paterson DH, Latimer AE, Rhodes RE, et al. New Canadian Physical Activity Guidelines. Applied Physiology, Nutrition and Metabolism. 2011;36:36–46.

4. DeFronzo RA, Ferrannini E. Insulin Resistance: A Multifaceted Syndrome Responsible for NIDDM, Obesity, Hypertension, Dyslipidemia, and Atherosclerotic Cardiovasculr Disease. Diabetes Care. 1991;14(3):173–94.

5. Thyfault JP, Krogh-Madsen R. Metabolic disruptions induced by reduced ambulatory activity in free-living humans. Journal of Applied Physiology. 2011 Oct;111(4):1218–24.

6. Colley RC, Garriguet D, Janssen I, Craig CL, Clarke J, Tremblay MS. Physical activity of Canadian adults: accelerometer results from the 2007 to 2009 Canadian Health Measures Survey. Health reports / Statistics Canada, Canadian Centre for Health Information. 2011 Mar;22(1):7–14.

7. Trost SG, Owen N, Bauman AE, Sallis JF, Brown W. Correlates of adults’ participation in physical activity: review and update. Medicine and science in sports and exercise. 2002 Dec;34(12):1996–2001.

8. Schnohr P, Marott JL, Jensen JS, Jensen GB. Intensity versus duration of cycling, impact on all-cause and coronary heart disease mortality: the Copenhagen City Heart Study. European journal of preventive cardiology. 2012 Feb;19(1):73–80.

9. Rosenkilde M, Auerbach PL, Reichkendler MH, Ploug T, Stallknecht BM, Sjödin A. Body fat loss and compensatory mechanisms in response to different doses of aerobic exercise - a randomized controlled trial in overweight sedentary males. American journal of physiology. Regulatory, integrative and comparative physiology. 2012 Aug 1;

10. Gibala MJ, McGee SL. Metabolic adaptations to short-term high-intensity interval training: a little pain for a lot of gain? Exercise and Sport Sciences Reviews. 2008 May;36(2):58–63.

11. Little JP, Safdar A, Wilkin GP, Tarnopolsky MA, Gibala MJ. A practical model of low-volume high-intensity interval training induces mitochondrial biogenesis in human skeletal muscle: potential mechanisms. The Journal of Physiology. 2010 Mar 15;588(Pt 6):1011–22.

12. Hood MS, Little JP, Tarnopolsky M a, Myslik F, Gibala MJ. Low-Volume Interval Training Improves Muscle Oxidative Capacity in Sedentary Adults. Medicine and science in sports and exercise. 2011 Mar 25;I:1849–56.

13. Little JP, Gillen JB, Percival M, Safdar A, Tarnopolsky M a, Punthakee Z, et al. Low-volume high-intensity interval training reduces hyperglycemia and increases muscle mitochondrial capacity in patients with type 2 diabetes. Journal of applied physiology (Bethesda, Md. : 1985). 2011 Aug 25;111(August):1554–60.

14. Trapp EG, Chisholm DJ, Freund J, Boutcher SH. The effects of high-intensity intermittent exercise training on fat loss and fasting insulin levels of young women. International journal of obesity (2005). 2008 Apr;32(4):684–91.

15. Metcalfe RS, Babraj J a, Fawkner SG, Vollaard NBJ. Towards the minimal amount of exercise for improving metabolic health: beneficial effects of reduced-exertion high-intensity interval training. European journal of applied physiology. 2011 Nov 29;112(7):2767–75.

16. Jakeman J, Adamson S, Babraj J. Extremely short duration high-intensity training substantially improves endurance performance in triathletes. Applied Physiology, Nutrition and Metabolism. 2012;981(May):976–81.

17. Gillen JB, Little JP, Punthakee Z, Tarnopolsky MA, Riddell MC, Gibala MJ. Acute high-intensity interval exercise reduces the postprandial glucose response and prevalence of hyperglycemia in patients with type 2 diabetes. Diabetes, obesity & metabolism. 2012 Jan 23;

18. Tarnopolsky M a, Pearce E, Smith K, Lach B. Suction-modified Bergström muscle biopsy technique: experience with 13,500 procedures. Muscle & Nerve. 2011 May;43(5):717–25.
